# Variance of *Zein* Protein and Starch Granule Morphology between Corn and Steam Flaked Products Determined Starch Ruminal Degradability Through Altering Starch Hydrolyzing Bacteria Attachment

**DOI:** 10.3390/ani9090626

**Published:** 2019-08-29

**Authors:** NingNing Xu, DiMing Wang, JianXin Liu

**Affiliations:** Institute of Dairy Science, MoE Key Laboratory of Molecular Animal Nutrition, College of Animal Sciences, Zhejiang University, Hangzhou 310058, China

**Keywords:** steam flaking, γ-*zein* protein, starch granule morphology, starch hydrolyzing bacteria

## Abstract

**Simple Summary:**

Previously, we found that starch ruminal degradation kinetics between raw and steam flaked corn were significantly different, but the mechanism is still ambiguous. Currently, we have demonstrated that γ-*zein* protein, encapsulating the starch granules into the hydrophobic starch–protein matrix, and starch granule characteristics were dramatically different between raw and steam flaked corns. Along with the totally different abundance and procedures of starch hydrolyzing bacteria attached to the two grain types, we concluded that γ-*zein* protein content and starch granule morphology of corn grain determined starch ruminal degradation kinetics through altering the abundance and procedure of starch hydrolyzing bacteria attached to corn grain in rumen.

**Abstract:**

The current study investigated differences of γ-*zein* protein contents and starch granule characteristics between raw and steam flaked corns and their influences on ruminal starch hydrolyzing bacteria (SHB) attached to corn grain. Two types of raw (Corn1 and Corn2) and their steam-flaked products (SFCorn1 and SFCorn2) were applied to explore physiochemical structures and SHB attachment. SDS-PAGE was conducted to detect γ-*zein* protein patterns, scanning electron microscope, and small angle X-ray scattering were performed to obtain starch granule morphology, while crystallinity, DQ starch, and DAPI staining were applied to quantify SHB. The steam flaking process destroyed γ-*zein* proteins and gelatinized starch granules. The median particle size of Corn1 and Corn2 starch granules increased from 17.8 and 18.0 μm to 30.8 and 26.0 μm, but crystallinity decreased from 22.0 and 25.0% to 9.9 and 16.9%, respectively. The percentage of SHB attached to Corn1 residues decreased (*p* = 0.01) after 4 h incubation, but SHB attached to SFCorn1 residues increased (*p* = 0.03) after 12 h incubation. Thus, the differences of γ-*zein* proteins and starch granule physiochemical structures between raw and steam flaked corn played an important role in improving the rate and extent of starch ruminal degradation through altering the process of SHB attached to corn.

## 1. Introduction

Starch granule is the basic unit of starch in corn endosperm and embedded in the starch–protein matrix [1,2,3], which influences the starch digestion by microbes. *Zein* protein, consisting of 4 subclasses (*α, β, γ*, and *δ*) during protein assembly, is the primary protein in the starch–protein matrix [4] and encapsulates starch granules into a hydrophobic matrice during corn maturity, with the *β-* and *γ*-*zeins* cross-linking and α- and *δ*-*zeins* penetrating the network. The destruction of the starch–protein matrix makes the starch granules easily accessible to amylolytic digestion by ruminal bacteria. Hoffman et al. (2011) found that the ensiling process destroyed the corn starch–protein matrix and contributed to greater access to starch granules [2]. In addition to the starch–protein matrix, granule morphology and size, the degree of granule crystallinity and extent of granule damage also influence starch digestibility [5]. Naguleswaran et al. [6] found that different granule morphology and crystallinity of triticale, wheat, and corn led to variance of the rate and extent of starch hydrolyzing, indicating that the natural characteristics of starch granules control starch amylolysis. 

The application of heat, moisture, and pressure during the steam flaking process improves cereal starch ruminal digestion by an average of 10% [7]. Our previous study also found that the rate and extent of starch ruminal degradation significantly increased after steam flaking [8], but little information is available on the mechanism by which steam flaking improves starch degradation kinetics. Cereal grain undergoes physical structure changes during steam flaking and the subsequent rolling process [9], and the high temperature during the steam flaking process could induce starch gelatinization [10] and denature proteins that form starch–protein matrix. Thus, the steam flaking process destructs the protection of starch granules and enhances the accessibility of amylose to starch granules. Ruminal starch hydrolysis is based on microorganisms activity [11], and the physiochemical structure differences between barley and corn altered starch hydrolysis through changing starch hydrolyzing bacteria (SHB) abundance [12]. Therefore, the objective of this study was to investigate how the destruction of starch–protein matrix and starch granule morphology via steam flaking process influenced starch ruminal degradation kinetics through altering the starch hydrolyzing bacteria attached to corn grain. 

## 2. Materials and Methods

### 2.1. Experimental Design and Chemical Profiles 

Two commercial corn samples (Corn1 and Corn2) and their steam flaked products (SFCorn1 and SFCorn2) were randomly collected from two different steam flaking plants to detect differences of physiochemical structures and the attachment of SHB between raw and steam flaked corns. SFCorn1 was soaked with water in a steel bin for 3.5 h, then steamed for 60 min under 106 °C, the flake thickness was 1.20 mm. SFCorn2 was soaked with water for 2 h, and then steamed at around 100 °C for 60 min, the flake thickness was 1.70 mm. Chemical compositions (Supplementary Table S1) are shown in our previous study [8]. γ-*zein* protein contents and starch granule characteristics, including granule morphology, size, and crystallinity, were obtained to describe the physiochemical structure differences between raw and steam flaked corns. A two-run in situ procedure was performed to obtain incubation residues, which were used to detect the abundance of SHB attached to corn residues.

### 2.2. Scanning Electron Microscope (SEM)

Starch granules were isolated according to Sandhu et al. [13]. Briefly, the sodium hydrogen sulfite steeped corn samples were ground with distilled water, then the slurry was screened through 100, 200, and 325 mesh nylon cloth successively. The final screened slurry was allowed to stand for 4–5 h and centrifuged at 2800 rpm for 5 min after suctioning the supernatant, then the white starch layer was collected. The sliced endosperm samples and isolated starch granules were mounted on circular aluminum stubs and coated with a thickness of 12 nm gold film, and then scanned and photographed in a SEM (TM-1000, Hitachi, Japan) at an accelerating voltage of 15 kV.

### 2.3. SDS-PAGE

*Zein* protein, which is hydrophobic, could hinge with starch granules into starch–protein matrix in corn endosperm, thus the *zein* protein contents in corn determine the rate and extent of starch degradation. An organic solvent was applied to extract *zein* proteins according to Liu et al. [14] to represent the starch–protein matrix differences between raw and steam flaked corns. In brief, 100 mg air dry corn samples were mixed with 1 mL of *zein* extraction buffer (70% [vol/vol] ethanol, 2% [vol/vol] 2-mercaptoethanol, 3.75 mM sodium borate [pH 10], 0.3% SDS) in a 2 mL tube (Axygen), and centrifuged at 15,700 × g for 20 min after 2 h of incubation at room temperature. Then 100 μL of the supernatant, along with an additional 10 μL of 10% [wt/vol] SDS solution, was transferred into a new tube and freeze-dried. Next, 100 μL distilled water was added to dissolve the protein. Finally, a mixture of 8 μL protein solution and 2 μL protein loading buffer was analyzed by SDS-PAGE gel (15% [wt/vol]) for γ-*zein* accumulation patterns.

### 2.4. Starch Granule Particle Size Analysis 

Starch granule particle size analysis was carried out using a BT-9300 (Bettersize instruments Ltd., China). The isolated starch granules were suspended in distilled water and stirred for 1 min. A general purpose analysis model was used to detect particle size, and 1.53 and 0.1 were chosen as particle refractive and absorption index, respectively. The refractive index of dispersant water was 1.33. The obscuration in all the measurements ranged from 10 to 20%. Particle size was described as 10th percentile [D(10)], median percentile [D(50)], and 90th percentile [D(90)] [15].

### 2.5. Small Angle X-Ray Scattering (SAXS)

The SAXS technique was applied to obtain crystallinity characteristics of isolated starch granules using a SAXS/WAXS SYSTEM (XENOCS) instrument with Cu Kα radiation of wavelength 1.5418 Å and operated at 50 kV and 60 mA, according to Shrestha et al. [16]. Samples were presented in 2 mm sealed quartz capillaries as suspensions, with a 1370 mm sample-to-detector distance. The diffraction intensity of 2θ angle was detected between 0º to 25º and scattering was measured for 600 s. Scattering data were background corrected and radially averaged, as reported previously [17]. The SAXS data was analyzed by MDI JADE 6.0 software. 

### 2.6. In Situ Procedures and Starch Hydrolyzing Bacteria Collection

The in-situ procedures were approved by the animal care committee at Zhejiang University (Hangzhou, China) and were in accordance with the guidelines for animal research.

Three rumen cannulated male Hu-sheep (body weight 35±5 kg, DM intake 1.5 kg) were applied to obtain in situ residues of Corn1 and SFCorn1, and the sheep were fed a basal diet containing 400 g concentrate (which mainly contained ground corn, cottonseed, soybean meal, and wheat bran) and Chinese wild rye ad libitum. Four g (dry matter) of coarsely ground corn samples were placed into each nylon bag (10 × 20 cm; 50 μm pore size; Ankom Technology Corp., Macedon, NY, USA) and tied at the end of nylon ropes, then put all the nylon bags in ventral sac of rumen in two separate runs (3 bags for each run) and incubated for 4, 12, 24, and 48 h based on an all in-gradual out schedule. The bacteria attached to the incubated residues were collected after removal from the rumen [12]. Briefly, the nylon bags were gently rinsed with 1 × PBS buffer and transferred the in situ residues to sterilized beakers, then mixed around 8 g of wet individual residues with 35 mL phosphate rinsing buffer, mechanically pummeled for 6 min, before filtering through 8 layers of cheesecloth. Four hundred μL filtered fluids were transferred to a 1.5 mL tube and centrifuged at 4500 × g for 10 min immediately, then the pellet was collected for the following detection. 

### 2.7. Quantification of Starch Hydrolyzing Bacteria

The relative abundance of SHB was determined by DQ starch staining combined with DAPI staining according to Xia et al. [12]. DQ starch staining was executed after collecting the bacteria attached to the incubated samples [18], and DAPI staining followed. Microscopy examination (Leica Microsystems CMS Gmbh) was performed to detect the DQ starch and DAPI staining, and 30 images of each stained slide were taken randomly. ImageJ software was applied to count the stained cell numbers [19]. The relative abundance of SHB was evaluated as DQ starch stained cell numbers/DAPI stained cell umbers in the whole scope. 

## 3. Results 

### 3.1. Zein Protein Pattern and Starch–Protein Matrix

**Figure 1:**
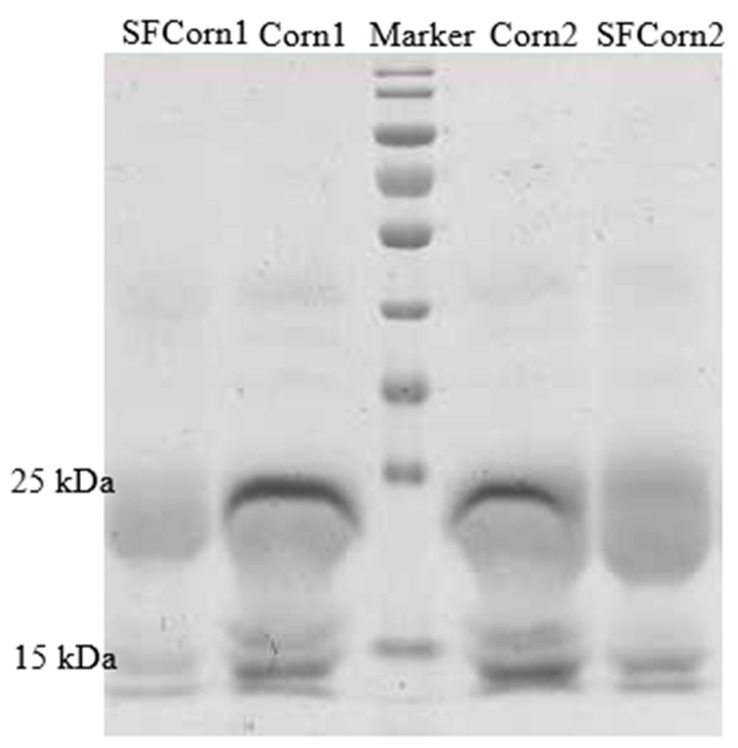
SDS/PAGE analysis of γ-*zein* proteins from equal amounts of endosperm flour of Corn1, steam (Figure 1) (SFCorn1), Corn2, and steam flaked Corn2 (SFCorn2). The identity of specific types of γ-*zein* proteins are shown on the left [2].

**Figure 2:**
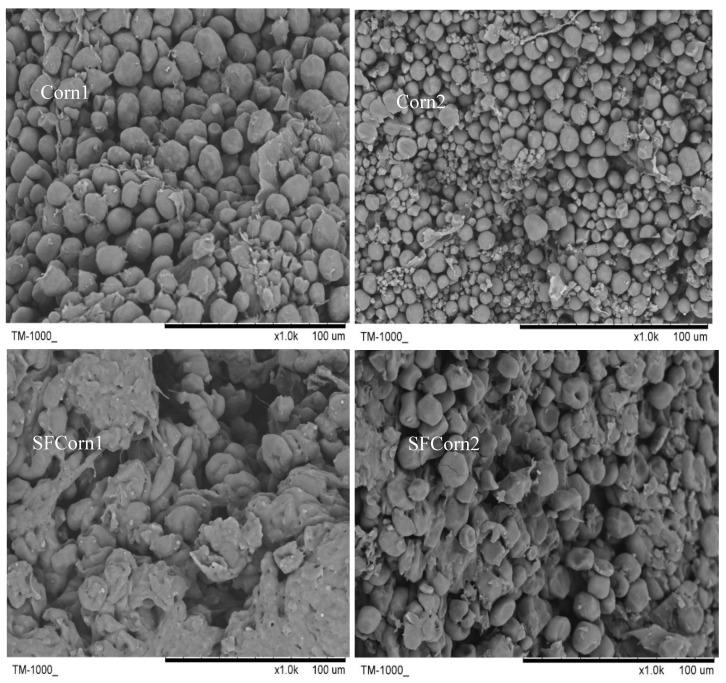
Scanning electron microscopic images (1000×) of Corn1, steam flaked Corn1 (SFCorn1), Corn2, and steam flaked Corn2 (SFCorn2).

The SDS-PAGE results indicated that abundances of 16 kDa and 27 kDa *γ*-*zein* proteins in Corn1 and Corn2 were notably greater than that in SFCorn1 and SFCorn2 (Figure 1), respectively, indicating that the hydrophobic starch–protein matrix was damaged dramatically in steam flaked corns. A paralogous 15 kDa *γ*-*zein* protein was also observed, except 16 kDa *γ*-*zein* protein currently, and both the 15 kDa and 16 kDa *γ*-*zein* proteins were lower in SFCorn1 and SFCorn2. The SEM images of endosperm (Figure 2) showed that the spherical starch granules were accumulated in the endosperm and some other chemicals (e.g., protein, fat, and fiber) penetrated into the granule accumulations and even encapsulated some granules, but more looser and irregular starch granule accumulations were observed in steam flaked products, indicating a more persistent starch–protein matrix and a more compacted starch granule accumulations in raw corn samples.

### 3.2. Starch Granule Morphology

**Figure 3:**
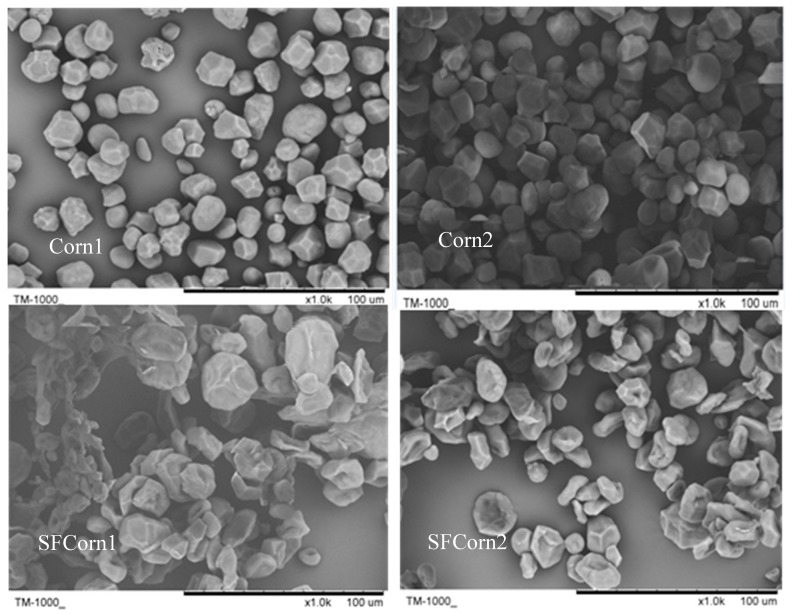
Scanning electron microscopic images (1000×) of isolated starch granules from Corn1, steam flaked Corn1 (SFCorn1), Corn2, and steam flaked Corn2 (SFCorn2).

The endosperm morphology of raw and steam flaked corns are displayed in Figure 2. The granules in steam flaked corns were gelatinized more intensely than those in raw corns. To detect any morphology changes of starch granules, SEM images of isolated granules were obtained and exhibited in Figure 3. Spherical and polygonal-shaped starch granules of various sizes were observed in raw corns, but shrunk into discoid after steam flaking. The starch granules of SFCorn1 pitted severely compared with that of SFCorn2, which could be due to the higher steaming temperature and lower flake density. The starch granule particle size distributions are displayed in Table 1. Starch granules were swelled due to high temperature and moisture during steam flaking, and the median particle sizes (D50) of starch granules were increased dramatically. The D50 of Corn1 and Corn2 were similar (17.77 vs.17.98 μm), but a greater D50 was observed in SFCorn1 (30.83 vs. 26.03 μm) after steam flaking.

### 3.3. Starch Granule Crystallinity

**Figure 4:**
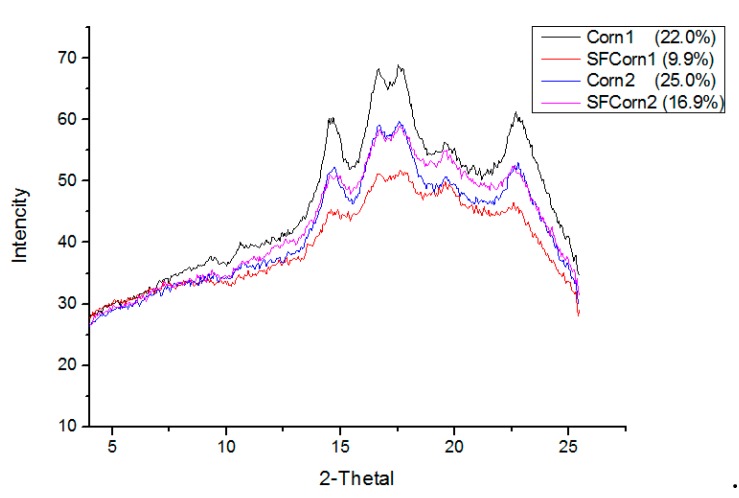
Small angle X-ray scattering profiles of Corn1, steam flaked Corn1 (SFCorn1), Corn2, and steam flaked Corn2 (SFCorn2).

The SAXS scattering patterns of raw and steam flaked starch granules show five characteristic peaks at approximate 2θ values of 14º, 17º, 18º, 20º, and 23º (Figure 4), which determine the granule crystal type. No remarkable differences in the position of characteristic peaks were observed between raw and steam flaked corns, indicating that starch granule crystal form was not altered by steam flaking. The absorbance intensities between Corn1 and SFCorn1 starch granules were prominently different, inducing the crystallinity of Corn1 decreased from 22.0 to 9.9%, but only a small difference (at around 2θ values of 18º, 20º, and 23º) was observed between Corn2 and SFCorn2, which led to a small decrease of crystallinity from 25.0 to 16.9%. 

### 3.4. Starch Hydrolyzing Bacteria Attached to Incubation Residues

Most grain starch is degraded in the rumen by micro-organisms, especially SHB, and the key process of starch degradation is the attachment of ruminal bacteria to corn. The bacteria attached to corn during rumen incubation, which is recorded in Table 2. The SHB percentages of Corn1 and SFCorn1 incubation residues were between 20–50%. The SHB relative abundances of Corn1 residues decreased significantly (*p* = 0.01) after 4 h incubation and stayed in a lower level, but an increase trend was observed before 48 h incubation. The SHB relative abundances of SFCorn1 residues were lower at the beginning of rumen incubation (*p* = 0.03) but increased dramatically after 12 h incubation and stayed in a high level until the end of incubation. Although the SHB relative abundances of 4 and 24 h incubation residues of Corn1 were greater (*p* < 0.01) and lower (*p* = 0.02) than that of SFCorn1, respectively, no difference was observed between Corn1 and SFCorn1 (*p* = 0.33, not show here), indicating that the process, but not the relatively abundance, of SHB attachment was significantly influenced by physiochemical structure differences.

## 4. Discussion

Our previous study indicated that the steam flaking process improved the rate and extent of starch degradation [8], and the different ruminal degradation kinetics with similar nutrients content between raw and steam flaked corn samples implied that the endosperm structures led to various rumen degradability [7]. Starch is the most important chemical substance in corn endosperm and consists of starch granules, which were crystallized by polysaccharides chains during endosperm synthesis [20]. Starch granules are encapsulated by prolamins into the starch–protein matrix in endosperm, which limits starch granules scattering during rumen fermentation and thus inhibits starch degradation [21]. A previous study showed that differences in digestion between isolated maize and barley starch granules were small [22], hence the starch–protein matrix might be more important in limiting microbial starch degradation than granule morphology [3]. 

Corn with a greater percentage of vitreous endosperm showed lower in vitro or in situ starch digestibility [23] due to the greater level of prolamin proteins [24], and starches in low protein content maize hydrolyzed faster than those starches in high protein contents maize [25]. Ruminal bacteria have inadequate ability to hydrolyze *zein* proteins, but the ruminal bacteria are expert in hydrolyzing starch granules, thus, the degradation of *zein* protein limited starch ruminal degradability [26,27]. Hoffman et al. [2] also found that the decreased *γ*-*zein*s during ensiling led to the increased ruminal starch degradation. The steam flaking process and the high-pressure during rolling broke down the bandage of matrix and scattered the granules into a disordered status, and, at the final stage, facilitating the availability of amylase to starch. In the present study, the lesser abundance of 16 kDa and 27 kDa *γ-zein* proteins in SFCorn1 and SFCorn2 indicated that *γ*-*zeins* were damaged under a high steaming temperature, thus, the destroyed *γ*-*zein*s currently contribute to the improvement of rate and extent of starch degradation. 

A starch granule is the basic unit of starch degradation in rumen and the granule structures play an important role in determining energy release during digestion. Thermomechanical treatments during steam flaking process could alter starch granule structures, thus improving starch digestibility [28]. Less intact spherical and polygonal-shaped starch granules of various sizes were observed in steam flaked corns, indicating that the steam flaking process mechanically damaged and apparently gelatinized starch granules. The morphology changes increased the granule surface, probably resulting in exposure of the starch chains to microorganisms, especially SHB, and thus increased the rate and extent of starch degradation in rumen. Starch granules were much larger in diameter in good quality flakes [9], which implied that the steam flaking process of Corn1 was better than that of Corn2. Small starch granule cereals had greater starch digestibility than larger starch granules cereals [29], which was not consistent with current results. One explanation could be that the current swollen granules with a larger granule size had a greater surface area and more porosity, which might attribute to the higher starch degradation. Dhital et al. (2010) also observed a positive correlation between digestion rate and granule surface area [15]. 

The swelling process leads to the increase of granule porosity and inflates semi-crystalline structure, which determines the crystallinity of starch granule. An amorphous fraction of a starch granule is easier to be swollen by high temperature and moisture, but the crystalline lamellae is more resistant to being swollen, thus, the crystalline region of Corn1 decreased more prominently than Corn2 due to the lower crystallinity. On the other hand, the rolling process after steam flaking destroyed crystalline lamellae structures, which enhanced the decrease of crystallinity. However, the similar characteristic peaks between raw and steam flaked corns suggested that the steam flaking process did not influence crystal form. The decreased crystallinity allows amylase to penetrate into granules easily, and in turn, increases the rate and extent of starch degradation, thus, granules with less dense or crystalline structures are easier to be digested by amylase [30]. The semi-crystalline core of raw starch granules decreased after the steam flaking process, which increased starch enzymatic degradation depending on accessibility of α-amylase to carbohydrate chains [31], thus, the decreased granule crystallinity in the present study contributed to the increase in the rate and extent of starch degradation.

Cell-surface-associated (CSA) α-enzymes, producing by bacteria attached to corn, played a dominant role in starch hydrolysis [32,33], thus, the various SHB abundance between Corn1 and SFCorn1 implied different starch enzymatic hydrolysis activities. The SHB abundance of Corn1 decreased after 4 h and kept in the low level during the following fermentation process, therefore, a lower CSA-enzymatic process was expected in Corn1. The lower SHB abundance of SFCorn1 in the beginning indicated that starch degradability of SFCorn1 was lower, the reason for this perhaps being that the Maillard reaction during steam flaking restricted attachment of SHB to SFCorn1. But the SHB abundance of SFCorn1 increased quickly after 12 h incubation, and the highest SHB abundance was observed in 24 h incubation residues, indicating that SFCorn1 is more accessible to SHB, thus increasing starch ruminal degradation. Most of the starch could be degraded in rumen, but resistant starch could not be hydrolyzed by SHB due to the intact starch–protein matrix. Leitch et al. [34] found that lots of *Ruminococcus bromii*, which was the main component of starch hydrolyzing bacteria, was enriched around the resistant starch in human feces and *Ruminococcus bromii* abundance increased as resistant starch enhanced in diets. Therefore, the increased SHB percentage of 48 h incubation residues in Corn1 implied that more resistant starch existed, indicating a lower starch degradability. On the contrary, the SHB abundance of SFCorn1 decreased after 24 h incubation due to the damage of the starch–protein matrix during the steam flaking process.

## 5. Conclusions

The variance of starch ruminal degradation between raw and steam flaked corn was mainly attributed to denature of *zein* proteins and the breakdown of the starch–protein matrix, augment of starch granules surface area, and decreasing the granule crystallinity, which could alter starch hydrolyzing bacteria attached to corn samples in rumen. These findings provided a new idea to evaluate the quality of steam flaking based on abundance of SHB attached to steam flaked products. 

## Figures and Tables

**Table 1:** Size distribution of starch granules from Corn1, steam flaked Corn1 (SFCorn1), Corn2, and steam flaked Corn2 (SFCorn2).

Sample	Mastersizer Data				
	D10	D25	D50	D75	D90
Corn1	11.15	14.29	17.77	21.70	26.97
SFCorn1	11.29	20.02	30.83	50.27	96.34
Corn2	11.24	14.45	17.98	24.44	27.15
SFCorn2	9.99	17.86	26.03	45.20	54.60

**Table 2:** Relative abundance of starch hydrolyzing bacteria (SHB) attached to Corn1 and steam flaked corn 1 (SFCorn1)^1^.

Sample	Incubation Time (h)				SEM	*P*
	4	12	24	48		
Corn 1, %	43.9^aA^	28.8^b^	20.1^bB^	31.5^b^	3.73	0.01
SFCorn 1, %	34.6^bB^	23.3^b^	44.9^aA^	35.7^a^	4.06	0.03

^1^ Means with different lowercase within the same row differ significantly (*p* < 0.05). Means with different capital letters within the same column differ significantly (*p* < 0.05).

## Supplementary Materials

The following are available online at https://www.mdpi.com/2076-2615/9/9/626/s1, Table S1: Chemical profiles of raw (RC) and steam flaked corn (SFC) from two commercial plants. % of DM.
