# Supplementary material for: Variance of Zein Protein and Starch Granule Morphology between Corn and Steam Flaked Products Determined Starch Ruminal Degradability Through Altering Starch Hydrolyzing Bacteria Attachment
Source: Animals (Basel). 2019 Aug 29;9(9):626. doi: 10.3390/ani9090626 (PMC6769831; doi:10.3390/ani9090626)
Supplement: Supplementary file 1 [file animals-09-00626-s001.pdf]

Table 1. Chemical profiles of raw (RC) and steam flaked corn (SFC) from two commercial plants. % of DM.

Item[1]	Plant1		Plant2	
	**Corn1**	**SFCorn1**	**Corn2**	**SFCorn2**
DM (%)	89.4	91.5	89.3	89.4
Starch	69.0	79.7	69.5	76.2
CP	10.3	8.7	10.0	8.7
EE	3.4	0.5	2.5	0.6
Ash	1.1	1.7	1.4	0.8
NDF	8.8	6.1	9.5	7.2
ADF	2.7	1.9	2.6	2.3
ADL	0.8	0.9	0.8	0.6

[1]DM, dry matter; CP, crude protein; EE, ethanol extract; NDF, neutral detergent fiber; ADF, acid detergent fiber; ADL, acid detergent lignin.
